# 79 Clinical profile of patients with myositis attending the Paediatric Rheumatology Clinic at Aga Khan University Medical College East Africa (Nairobi); a case series. Autoimmune diseases

**DOI:** 10.1093/rheumatology/keac496.075

**Published:** 2022-10-07

**Authors:** Joan Ahimbisibwe, Minsari Ogega, Angela Migowa

**Affiliations:** Department of Paediatrics and Child Health, Aga Khan University Medical College East Africa (Nairobi)

## Abstract

**Background:**

Myositis comprises of a large group of inflammatory muscle conditions characterized by muscle pain and weakness including but not limited to juvenile dermatomyositis and post viral myositis. Juvenile dermatomyositis (JDM) is a systemic capillary vasculopathy characterized by proximal muscle weakness, skin and systemic manifestations. Post viral myositis is a condition characterized by acute onset of muscle pain, tenderness and weakness which occurs shortly after a viral illness. Both these conditions have been categorized as rare and literature is scarce in Sub Saharan Africa.

**Objective:**

To describe the clinical profile of patients with myositis in our setting.

**Methods:**

Retrospective chart review of children presenting with myositis attending the pediatric rheumatology clinic at AKUH, Nairobi, Kenya.

**Results:**

A total of 8 cases were reviewed, 5 males and 3 females with a median age of 8 years who presented between 2017 and 2022. Four had post viral myositis (male to female ratio 3:1) and four had JDM (male to female ratio 1:1). All patients with post viral myositis presented with difficulty walking, calf pain, history of an upper respiratory tract illness 2 weeks prior and laboratory findings of elevated creatinine kinase (CK). They were managed with analgesics and all had spontaneous resolution of symptoms within a month of presentation. The patients with JDM all presented with skin lesions and muscle weakness. All 4 had gottrons papules, 2 had a heliotrope rash and 1 developed calcinosis while on follow up. Only one had elevated level of CK. Anti-nuclear antibodies were positive in two of three patients tested. Myositis profile done in 2 of the patients showed positive anti-TIF1 antibodies in one while the other tested positive for anti-RO-52 antibodies and anti-NXP2 antibodies. Three patients had significant findings on echocardiography including mild aortic, tricuspid and mitral regurgitation, mitral stenosis and mitral valve prolapse. All the patients had a normal ejection fraction. Two patients had features of interstitial lung disease. One of the patients was in critical care for 3 months where he was pulsed with methylprednisone before transfer to an alternative facility where he eventually passed on. Two of the patients are on follow up at the clinic, receiving methotrexate and mycophenolate mofetil respectively in addition to prednisone. They have both had improvement of symptoms evidenced by an increase in the Childhood Myositis Assessment Score (CMAS) from 17 to 33 in 6 weeks and 41–52 in 6 months respectively. One of the patients was lost to follow up due to financial constraints.

**Conclusion:**

JDM and post viral myositis are not uncommon and risk being misdiagnosed especially in resource constrained environments. It is important for clinicians to be aware of the clinical presentation so as to develop a high index of suspicion hence prompting appropriate and timely care. Close follow up and counselling in appropriate clinics are crucial in the management of these patients but this may be a challenge due to financial constraints

